# Household Health Care Payments Under Rate Setting, Spending Growth Target, and Single-Payer Policies

**DOI:** 10.1001/jamahealthforum.2024.1932

**Published:** 2024-06-30

**Authors:** Jodi L. Liu, Federico Girosi, Ruolin Lu, Christine Eibner

**Affiliations:** 1 RAND, Santa Monica, California

## Abstract

**Importance:**

Households have high burden of health care payments. Alternative financing approaches could reduce this burden for some households.

**Objective:**

To estimate the distribution of household health care payments across income under health care reform policies.

**Design, Setting, and Participants:**

Cross-sectional study with microsimulation used nationally representative data of the US population in 2030. Civilian, noninstitutionalized population from the 2022 Current Population Survey linked to expenditures from the 2018 and 2019 Medical Expenditure Panel Survey and 2022 National Health Expenditure Accounts were included.

**Exposure:**

Rate regulation of hospital, physician, and other health care professional payments equal to the all-payer mean in the status quo, spending growth target at 4% annual per capita growth, and single-payer health care financed through taxes.

**Main Outcomes and Measures:**

Household health care payments (out-of-pocket expenses, premiums, and taxes) as a share of compensation.

**Results:**

The synthetic population contained 154 456 records representing 339.5 million individuals, with 51% female, 7% Asian, 14% Black, 18% Hispanic White, 56% non-Hispanic White, and 5% other races and ethnicities (American Indian or Alaskan Native only; Native Hawaiian or other Pacific Islander only; and 2 or more races). In the status quo, mean household health care payments as a share of compensation was 24% to 27% (standard error [SE], 0.2%-1.2%) across income groups (median [IQR] 22% [4%-52%] below 139% of the federal poverty level [FPL]; 21% [4%-34%] for households above 1000% FPL [11% of the population]). Under rate setting, mean (SE) payments by households above 1000% FPL increased to 29% (0.6%) (median [IQR], 22% [6%-35%]) and decreased to 23% to 25% for other income groups. Under the spending growth target, mean (SE) payments decreased from 23% to 26% (SE, 0.2%-1.2%) across income groups. Under the single-payer system, mean (SE) payments declined to 15% (0.7%) (median [IQR], 4% [0%-30%]) for those below 139% FPL and increased to 31% (0.6%) (median [IQR], 23% [3%-39%]) for those above 1000% FPL. Uninsurance fell from 9% to 6% under rate setting due to improved Medicaid access, and to zero under the single-payer system.

**Conclusions and Relevance:**

Single-payer financing based on the current federal income tax schedule and a payroll tax could substantially increase progressivity of household payments by income. Rate setting led to slight increases in payments by higher-income households, who financed higher payment rates in Medicare and Medicaid. Spending growth targets reduced payments slightly for all households.

## Key Points

**Question:**

What is the distribution of US household health care payments as a share of compensation under rate regulation, spending growth target, and single-payer policies?

**Findings:**

In this cross-sectional microsimulation analysis, single-payer health care financed by income and payroll taxes made mean payments more progressive, decreasing from 27% to 15% of compensation for the lowest-income households and increasing from 27% to 31% for the highest-income households. Modest rate setting benefitted lower-income households due to slightly improved access, while spending growth targets reduced payments slightly for all households.

**Meaning:**

Health care reforms can have heterogenous changes for households by income.

## Teaser

This cross-sectional microsimulation analysis simulates how overall health care payments and who pays for health care would change under rate regulation, spending growth target, and single-payer health care alternatives compared with the status quo.

## Introduction

Total US health care spending hit $4.5 trillion in 2022, a 29% increase over 10 years, after adjusting for inflation.^1^ The continued, large-scale spending growth underscores an urgent need for policy changes that will stem this tide. Recent proposals to curb spending growth include rate regulation with Medicare prices as a benchmark, global budgets, and spending growth targets.^2,3^ Several of these options have been implemented on a limited basis. For example, at least 8 states have implemented health care spending growth targets that aim to limit future increases.^4^ Other proposals pair rate regulation with coverage expansions and changes to health care financing, such as single-payer health care or Medicare For All proposals.^5,6^

Previous studies have found that—if implemented nationally—these types of proposals have the potential to reduce total health care spending.^7,8,9^ Although an implicit goal of many health reforms aimed at reducing spending is to make health care more equitable, few studies examine how proposed policies affect the distribution of who pays for health care. The burden of health care payments often make health care unaffordable for lower-income households facing large out-of-pocket (OOP) expenses, although health care payments became less regressive by income following the Affordable Care Act.^10,11,12^ Policy analyses should consider all forms of payment, including those that are evident, such as OOP and individual premium payments, and those that are less visible, such as employer premium contributions and tax payments that support health care programs.

In this study, we simulated how overall health care payments and who pays for health care would change under rate regulation, spending growth target, and single-payer health care alternatives compared with the status quo.

## Methods

RAND’s Human Subjects Protection Committee approved this study and waived informed consent because the study was not considered human participants research. This study followed the Strengthening the Reporting of Observational Studies in Epidemiology (STROBE) reporting guidelines for cross-sectional studies.

### Statistical Analysis

We conducted the simulation and statistical analyses using R statistical software (version 4.3.1; the R Project for Statistical Computing). We ran TAXSIM35 using the usincometaxes package in R. Standard errors (SEs) were bootstrapped and SEs calculated using the boot package in R, with resampling and 5000 replicates.

### Synthetic Population

Because no single data source contains the necessary information for assessing all health care payments, we constructed a synthetic population, using an approach similar to Carman et al.^10^ eTable 1 in Supplement 1 shows key model inputs and assumptions.

The civilian, noninstitutionalized population was from the RAND COMPARE model, which is a microsimulation of individuals and households that iteratively make insurance coverage decisions based on utility maximization and calculated premiums.^13^ We linked health expenditures from the 2018 and 2019 Medical Expenditure Panel Survey (MEPS) to income and household composition information in the 2022 Current Population Survey (CPS) Annual Social and Economic Supplement (ASEC) by matching records based on age, sex, race and ethnicity, health status, insurance category, and income. Similarly, we matched workers in the CPS-ASEC to firms in the 2019 KFF Employer Health Benefits Survey (EHBS) based on Census region, size, industry, employer offer status, and unionization status.

We added institutionalized and active-duty military populations from the 2022 American Community Survey (ACS) Public Use Microdata Sample (PUMS). We adjusted health expenditures from the MEPS to match amounts reported in the 2022 National Health Expenditure Accounts (NHEA) by service type and payer. We aligned income and wages to totals from the Bureau of Economic Analysis^14^ by increasing income and wages for individuals with top-coded values in the CPS by a fixed percentage. We adjusted household income and wages proportionally to match the household income and wage distributions by quintiles reported by the Congressional Budget Office.^15^

To inflate the synthetic population to 2030, we used the 2023 national population projections by age, race and ethnicity, and sex from the Census Bureau.^16^ We inflated income and wages using the Consumer Price Index for Urban Consumers (CPI-U).^17^ For health care expenditures, we used per capita growth rates by payer from NHEA.

### Microsimulation of Insurance Coverage and Premiums

We used a utility maximization approach to estimate how individuals would make health insurance enrollment decisions (eMethods 1 in Supplement 1). We estimated premiums based on mean expenditures of people enrolled in each insurance risk group. We calibrated the model to ensure that outputs under baseline policy conditions reflected observed enrollment (by coverage type) and premiums.

When modeling rate setting, we adjusted expenditures for each insurance type by multiplying by the ratio of the target payment rates to status quo rates relative to Medicare for that insurance type.

Under a single-payer system, we assumed that increased demand for services (from newly insured individuals and more generous coverage) may not be fully met. The extent of unmet care would depend on hospitals, physicians, and other health care professionals’ willingness and ability to provide services based on payment levels and other factors. Based on prior analyses, we assumed that unmet demand would approximately equal 50% of the new demand under a single-payer plan with hospital, physician, and other health care professional payment rates equal to the all-payer mean in the status quo.^8,18^ We assumed supply for prescription drugs and medical equipment was unconstrained.

### Health Reform Policies

We modeled 4 policy scenarios (Table 1). The first represents the status quo, or US health insurance policies that will be in place in 2030 given current laws.

**Table 1.** Policy Scenarios

Scenario	Description	Insurance plans available	Provider payment^a^	Prescription drug prices	Administrative cost	Per capita expenditure growth rate
Status quo	Current policies carried forward to 2030, without temporary enhancements to premium tax credits that expire in 2025 under the Inflation Reduction Act and without other significant policy changes (eg, related to Medicaid expansion in additional states, Medicaid eligibility, and the tax exclusion of employer-sponsored insurance)	Employer, nongroup, Medicaid, Medicare, other public	No change in current levels	No change in current levels	No change in current levels	Projected growth in the National Health Expenditure Accounts
Rate setting	All-payer rate setting with provider payment equal to the all-payer mean across all service types in the status quo	Same as status quo	110% of Medicare rates	Same as status quo	Same as status quo	Same as status quo
Spending growth target	Cap on annual growth in per capita health care spending starting in 2025	Same as status quo	Same as status quo	Same as status quo	Same as status quo	4%
Single payer	Public plan administered by the federal government or third-party administrator; financed by a federal income tax and a payroll tax, Medicare payroll tax allocated to the single-payer program, and state maintenance of effort equal to state Medicaid funds in the status quo	98% Actuarial value plan available to all US residents (including undocumented immigrants)	110% of Medicare rates	90% of Medicare rates	7%	Same as status quo

^a^ Provider payment refers to health care payments made to hospitals, physicians, and other health care professionals.

Our second scenario involves a rate setting approach in which hospital, physician, and other health care professional payments for all health care services reflect the all-payer mean under current law. Based on relative hospital, physician, and prescription drug prices in the literature, we estimated a status quo all-payer mean of 110% of Medicare rates across all services.^19,20,21,22,23,24,25^ This rate setting increased payment for Medicaid and Medicare, while decreasing payment for private insurance. This change has implications for taxpayer spending and for utilization because we assumed that higher payment levels lead to increased health care access among Medicaid enrollees.

Our third scenario is a spending growth target similar to programs implemented in Massachusetts, Delaware, Rhode Island, and other states. These programs set an annual, statewide global budget based on historical health care spending plus a percentage increase, typically ranging from 3% to 3.6%.^4^ States have various means to enforce the targets, such as public reporting, fines, and antitrust enforcement; however, these mechanisms have rarely been used.^26,27,28^ Nonetheless, per capita total health care expenditures were within 0.4% to 1.2% of the benchmark in Massachusetts from 2013 to 2019.^26^ To reflect imperfect enforcement, we set the annual growth rate for per capita spending starting in 2025 to 4%, which is above state benchmarks and below the status quo NHE projected annual growth of 4.8% to 5.2% between 2025 and 2030.^29^

In our single-payer scenario, we assumed hospital, physician, and other health care professional payment also equaled the all-payer mean of 110% of Medicare rates under current law and prescription drug and medical equipment prices were 90% of Medicare rates. We assumed the plan would have 98% actuarial value and a 7% administrative load, similar to the mean load across Medicare fee-for-service and Medicare Advantage under current law.^29^ We assumed hospital, physician, and other health care professional administrative costs would be lower, with costs for billing and insurance-related activities reduced by 32.6%,^30,31^ and no change to other administrative costs, such as general overhead and quality measurement costs.

### Household Health Care Payments

We examined household health care payments made by the civilian, noninstitutionalized population in the following categories: OOP payments, premium contributions (individual and employer), taxes supporting health care programs, and other (eMethods 2 in Supplement 1). In the status quo, these payments sum to the total NHE amount (less OOP payments for nondurable medical products that we excluded from the analysis). OOP payments include cost sharing in health insurance plans and spending for services not covered by insurance. We used the National Bureau of Economic Research TAXSIM program (version 35) to estimate federal personal income, state personal income, and payroll taxes, and allocated publicly funded expenditures to individuals in the model in proportion to relevant taxes.

The main outcome was household health care payments as a share of compensation. The numerator contained OOP payments, premiums, taxes supporting health care, and other payments for health care. The denominator contained household total income (wage, salary, and unearned income) and employer premium contributions because we assumed the incidence of employer premiums falls on workers in the form of forgone wages. In the single-payer scenario, household compensation also included wages passed back from employers that no longer paid premiums (eMethods 3 and eTable 2 in Supplement 1). We reported the mean, median, and IQR of household payments as a share of compensation and whether households paid more, less, or about the same using this metric under the reforms relative to the status quo. We reported these outcomes by 4 household income groups (without employer premium contributions) relative to the federal poverty level (FPL) in the status quo: below 139%, 139% to 400%, 401% to 1000%, and above 1000% FPL. Approximately 11% of the population were in households with income above 1000% FPL.

## Results

### Health Insurance Coverage

The synthetic population contained 154 456 records representing 339.5 million individuals, with 51% female, 7% Asian, 14% Black, 18% Hispanic White, 56% non-Hispanic White, and 5% other races and ethnicities (American Indian or Alaskan Native only; Native Hawaiian or other Pacific Islander only; and 2 or more races). Rate setting to the all-payer mean resulted in increased Medicaid enrollment (70.7 million vs 66.0 million in 2030) owing to higher public hospital, physician, and other health care professional payments that may contribute to better access to care (Table 2). For private insurance, lower hospital, physician, and other health care professional payments contributed to lower premiums, which increased nongroup enrollment to 23.5 million (compared with 17.9 million in the status quo) in 2030. The number of uninsured decreased from 29.7 million (9% of the population) in the status quo to 21.8 million (6% of the population). With the lower growth in health care spending in the spending growth target scenario, there was little change in overall enrollment. Under the single payer system, all individuals were on the single-payer plan, with no one uninsured.

**Table 2.** Demographic Characteristics and Estimated Health Insurance Enrollment, 2030^a^

Characteristic	Weighted participants, No. in millions (%)			
	Status quo	Rate setting	Spending growth target	Single payer
Primary health insurance				
Employer	158.4 (47)	157.0 (46)	158.8 (47)	0
Nongroup	17.9 (5)	23.5 (7)	16.8 (5)	0
Medicaid	66.0 (19)	69.7 (21)	66.2 (19)	0
Medicare	60.7 (18)	60.7 (18)	60.7 (18)	0
Other public	6.9 (2)	6.9 (2)	6.9 (2)	0
Single payer	0	0	0	339.5 (100)
Uninsured	29.7 (9)	21.8 (6)	30.2 (9)	0

^a^ This table reports data for the US civilian, noninstitutionalized population. The health care expenditures contain all service categories in the National Health Expenditure Accounts except for other nondurable medical products.

### Total Health Care Expenditures

**Figure 1.**
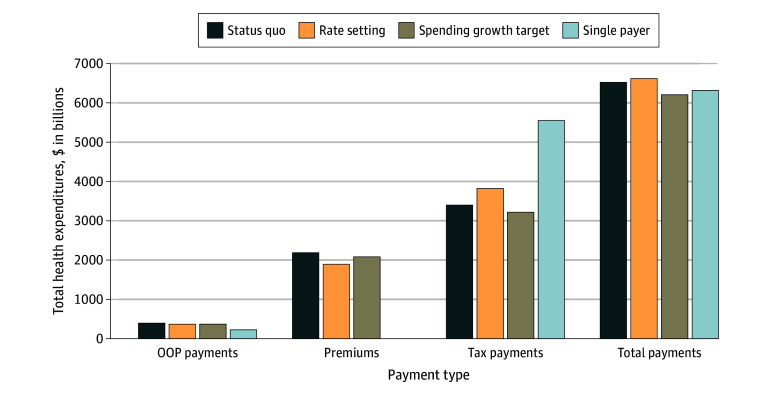
Estimated Total Health Care Expenditures by Type of Payment in 2030 Total payments also include a category of other payments, which we assumed were constant ($540 billion) across scenarios. OOP indicates out of pocket.

Figure 1 shows health care expenditures, overall and by payment type. The slightly higher total expenditures in the rate setting scenario relative to the status quo (2% higher) were due to more people being insured; mean spending per insured individual was slightly lower with rate setting ($20 836 vs $21 045). Although total expenditures under the single-payer system were relatively similar to the status quo (3% lower), mean spending per insured individual was much lower ($18 598 [12%] lower). With the spending growth target, there was a 5% decrease in total expenditures relative to the status quo. eFigure 1 in Supplement 1 shows estimated total health care expenditures under alternative assumptions.

Across types of payments, the largest change from the status quo occurred in the single-payer scenario with the shift to tax-financed coverage and lower OOP payments. With rate setting, there was some shift from OOP and premium payments to tax payments as hospital, physician, and other health care professional payments decreased for private insurers and increased for public payers. In the spending growth target scenario, there were small reductions in all payment types.

### Distribution of Household Health Care Payments by Income

**Figure 2.**
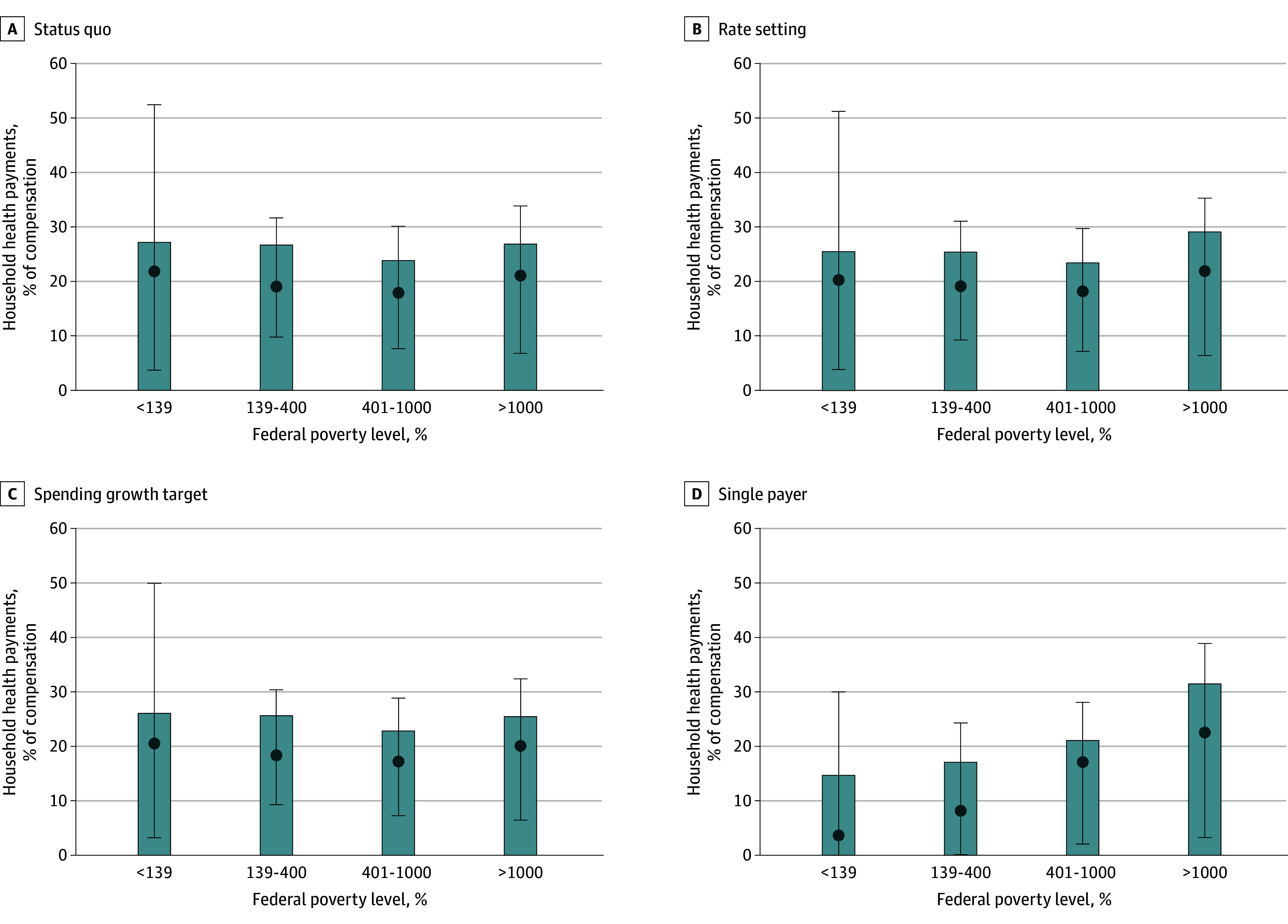
Household Total Health Care Payments as a Share of Compensation Payments made by the civilian, noninstitutionalized population. The x-axis shows household income as a percentage of the federal poverty level (FPL) in the status quo. Household compensation contains household income and employer premium contributions. The bars show mean payments, the dots show median payments, and the error bars show the IQR.

Figure 2 shows household health care total payments as share of compensation (mean, median, and IQR) by income group. In the status quo, mean (SE) household total payments as a share of compensation were similar across the income groups (24%-27% [0.2%-1.2%]). However, there was substantial variability in household payments within each income group, particularly for those with income below 139% FPL (median [IQR], 21.8% [3.7%-52.4%]).

Rate setting and the spending growth target resulted in small changes in the distribution of household payments across income compared with the status quo. With rate setting, mean payments for households with income below 1000% FPL decreased (−2% to −6% relative to the status quo), whereas mean payments for households with income above 1000% FPL increased by 9% because of higher hospital, physician, and other health care professional payments to public payers financed by taxes that disproportionately fall on higher-income households. With the spending growth target, there was a 4% to 5% decrease in mean total household payments for all income groups.

With single-payer financing, there was a dramatic change in the distribution of household payments. For households with incomes below 1000% FPL, this scenario resulted in lower mean payments relative to the status quo and less variability across households. Mean (SE) payments were 14.7% (0.7%) of compensation (median [IQR], 3.6% [0%-30.0%]) for those with incomes below 139% FPL and 21.1% (0.2%) (median [IQR], 17.1% [2.1%-28.1%]) for those with incomes between 401% and 1000% FPL. For households with incomes above 1000% FPL, payments as a share of compensation increased substantially to a mean (SE) of 31.5% (0.6%); however, variability remained large for this group (median [IQR], 22.5% [3.2%-38.9%]). eFigure 2 in Supplement 1 shows results under alternative financing assumptions including more and less progressive options.

Table 3 shows the mean and median payments as a share of compensation by payment type. In the status quo, mean OOP payments as a share of compensation decreased with increasing income. Below 1000% FPL, premium contributions were the largest portion of total payments on average. Tax payments for health care increased with income, reflecting the progressivity of tax schedules. Above 1000% FPL, mean tax payments were by far the largest portion of total payments as a share of compensation. In the rate setting scenario, there was a small shift from premium to tax payments relative to the status quo. In the spending growth target scenario, the types of payments were similar to those in the status quo. In the single-payer scenario, payments shifted primarily to tax payments, with relatively small OOP payments remaining and no premiums. As with the other scenarios, tax payments, based largely on existing progressive tax schedules, increased with income.

**Table 3.** Household Health Care Payments as a Share of Compensation, by Type of Payment and Income

Variable	Household health payments, % of compensation			
	OOP	Premium	Tax payments to health care	Total health care payments
**Status quo**				
<139% FPL				
Mean (SE)	6.4 (0.4)	12.0 (0.6)	8.2 (0.4)	27.2 (1.2)
Median (IQR)	0.7 (0-9.4)	0 (0-1.4)	0 (0-14.8)	21.8 (3.7-52.4)
139% to <400% FPL				
Mean (SE)	3.3 (0.1)	15.9 (0.2)	6.6 (0.2)	26.6 (0.2)
Median (IQR)	0.4 (0-3.1)	0 (0-17.8)	5.7 (0.9-12.5)	19.0 (9.8-31.6)
400% to <1000% FPL				
Mean (SE)	1.7 (<0.1)	11.9 (0.1)	8.7 (0.1)	23.8 (0.2)
Median (IQR)	0.3 (0-1.7)	3.5 (0-15.4)	6.1 (3.7-10.8)	17.9 (7.6-30.1)
≥1000% FPL				
Mean (SE)	0.4 (<0.1)	3.3 (0.1)	19.8 (0.4)	26.8 (0.5)
Median (IQR)	0.1 (0-0.7)	1.4 (0-8.2)	10.7 (3.5-19.8)	21.1 (6.8-33.8)
**Rate setting**				
<139% FPL				
Mean (SE)	6.5 (0.4)	10.3 (0.5)	8.1 (0.5)	25.5 (1.2)
Median (IQR)	0.6 (0-9.0)	0 (0-7.2)	0 (0-14.0)	20.3 (3.8-51.2)
139% to <400% FPL				
Mean (SE)	3.2 (0.1)	14.3 (0.1)	7.0 (0.2)	25.4 (0.2)
Median (IQR)	0.4 (0-2.9)	4.1 (0-17.8)	5.4 (1.8-11.9)	19.1 (9.3-31.1)
400% to <1000% FPL				
Mean (SE)	1.6 (<0.1)	10.3 (0.1)	10.0 (0.2)	23.4 (0.2)
Median (IQR)	0.3 (0-1.5)	3.8 (0-13.2)	6.5 (3.5-12.1)	18.2 (7.2-29.7)
≥1000% FPL				
Mean (SE)	0.4 (<0.1)	2.9 (0.1)	22.6 (0.5)	29.1 (0.6)
Median (IQR)	0.1 (0-0.6)	1.4 (0-6.9)	12.2 (3.3-22.3)	21.9 (6.4-35.3)
**Spending growth target**				
<139% FPL				
Mean (SE)	6.0 (0.4)	11.7 (0.6)	7.9 (0.4)	26.0 (1.2)
Median (IQR)	0.6 (0-8.8)	0 (0-1.5)	0 (0-14.1)	20.5 (3.2-49.9)
139% to <400% FPL				
Mean (SE)	3.1 (0.1)	15.3 (0.2)	6.3 (0.2)	25.6 (0.2)
Median (IQR)	0.4 (0-2.9)	0 (0-17.6)	5.5 (1.0-11.9)	18.4 (9.3-30.4)
400% to <1000% FPL				
Mean (SE)	1.6 (<0.1)	11.3 (0.1)	8.4 (0.1)	22.8 (0.2)
Median (IQR)	0.3 (0-1.6)	3.4 (0-14.6)	5.8 (3.5-10.3)	17.2 (7.3-28.9)
≥1000% FPL				
Mean (SE)	0.4 (<0.1)	3.1 (0.1)	18.7 (0.4)	25.5 (0.5)
Median (IQR)	0.1 (0-0.7)	1.3 (0-7.7)	10.2 (3.3-18.9)	20.1 (6.4-32.4)
**Single payer**				
<139% FPL				
Mean (SE)	4.6 (0.3)	0	9.5 (0.5)	14.7 (0.7)
Median (IQR)	0.5 (0-6.9)	0	0 (0-3.8)	3.6 (0-30.0)
139% to <400% FPL				
Mean (SE)	1.9 (0.1)	0	14.2 (0.2)	17.1 (0.2)
Median (IQR)	0.2 (0-1.4)	0	2.3 (0-21.5)	8.1 (0.1-24.3)
400% to <1000% FPL				
Mean (SE)	0.9 (<0.1)	0	18.6 (0.2)	21.1 (0.2)
Median (IQR)	0.1 (0-0.7)	0	14.8 (0.8-25.2)	17.1 (2.1-28.1)
≥1000% FPL				
Mean (SE)	0.2 (<0.1)	0	28.2 (0.5)	31.5 (0.6)
Median (IQR)	0.1 (0-0.3)	0	20.1 (2.4-35.0)	22.5 (3.2-38.9)

Abbreviations: FPL, federal poverty level; OOP, out of pocket.

With substantial variability in payments within the income groups, there were some households who paid more and some who paid less in each scenario relative to the status quo. Payment reductions in the rate setting and single-payer scenarios were more concentrated in the middle-income groups, whereas payment reductions from the spending growth target were more concentrated in higher-income groups (eFigure 3 in Supplement 1).

## Discussion

Across household income in the status quo, we estimated relatively similar mean household health care payments, ranging from 24% to 27% of compensation in the reported income groups. This represented a high burden of payments, especially for lower-income households. Although payments as a share of compensation was similar, absolute dollar amounts contributed by higher-income households were much greater than amounts contributed by lower-income households. Differences by payment type were larger, with lower-income households paying a larger share OOP and for premiums, and higher-income households paying more in taxes. One factor that contributed to these results was that about 9% of people with household incomes below 139% FPL had employer coverage in our analysis, and thus faced substantial premium incidence given our assumption that workers bear the cost of employer premium contributions. OOP payments can also be high for those with employer-sponsored insurance, given longstanding trends toward high cost sharing.^32^

We found relatively small changes in the distribution of household payments in the rate setting and spending growth target scenarios, with means of 23% to 29% and 23% to 26% of compensation, respectively. Although both policies reduced mean payments for households with incomes below 1000% FPL, the rate setting scenario also improved access for low-income households due to higher Medicaid payments. Better access came at the cost of higher tax payments among people with incomes above 1000% FPL. Spending growth targets did not improve access but reduced payments for all households, with larger reductions for high-income households.

In addition to covering all US residents and reducing OOP payments, the single-payer system dramatically changed who paid for health care in its shift to tax financing. Based on the progressivity of current income tax schedules and a flat payroll tax, we estimated decreases in mean health care payments for households with incomes below 1000% FPL, which represents nearly 90% of the population. For households with incomes above 1000% FPL, mean payments would rise, with larger increases for those with higher incomes. Below 139% FPL, we estimated a sizeable decrease in mean payments, but OOP payments were still nearly 5% of compensation. Policymakers designing a single-payer system could consider alternative tax schedules, such as caps for income above a certain threshold, or a minimum taxable income threshold to protect lower-income households. However, any reduction in tax payments would need to be balanced by increases in tax levels for other groups, or reductions in benefit generosity.

In terms of insurance coverage, the single-payer scenario by design covered the entire population. A possible incremental step toward a single-payer system could be to first establish rate setting, which reduced the uninsurance rate from 9% in the status quo to 6% with hospital, physician, and other health care professional payments equal to the all-payer mean.

### Limitations

This analysis had several limitations that should be considered when interpreting results. First, we may have introduced inaccuracies when we linked records across multiple datasets to construct the synthetic population. To mitigate against this concern, we ensured that aggregate statistics, such as population totals and national health spending, matched external benchmarks.

To model health care reforms, we made broad assumptions about design and implementation, including assumptions about how payment rates would change and how reforms would be financed. We based our assumptions on past policy examples. However, specific proposals may differ from what we have considered. For example, single-payer financing with different tax schedules or other taxes (eg, unearned income, sales, or value-added) would have very different incidence on households than what we estimated.

Our analysis did not account for noncompliance or tax avoidance. Although we assumed that hospitals, physicians, and other health care professionals reduced supply in response to lower payment rates, the magnitude of the response is difficult to anticipate and may diverge from our assumptions.

Finally, we assigned all tax incidence to current taxpayers, without taking deficit spending into account. Deficit spending must accrue to taxpayers. However, the timing is uncertain, and the incidence may depend on unmodeled factors, such as changes in tax structures over time.

## Conclusions

Under current US law, we estimated that the lowest-income households spend a sizeable portion of their income on health care. Although people with higher incomes pay more in absolute terms, as a percentage of income, the gradient was relatively flat. A tax-financed single-payer system could substantially shift the distribution of household health care payments, reducing mean payments for those with low incomes while increasing it for those with high incomes. Compared with the single-payer scenario, the other reforms had smaller impacts. However, savings from spending growth targets accrued primarily to higher-income households, whereas rate setting policies that raise Medicaid rates may improve access for lower-income households.

## Notes

Supplement 1. eMethods 1. RAND COMPARE Microsimulation Model eMethods 2. Adjustments to Health Care Expenditures and Payments eMethods 3. Calculating the Wage Pass Back eTable 1. Key Model Inputs and Assumptions eTable 2. Demographic Characteristics of the Synthetic Population, 2030 eTable 3. Estimated Wages in the Status Quo and Single-Payer Scenarios, 2030 eFigure 1. Estimated Total Health Care Expenditures Relative to the Status Quo under Alternative Assumptions eFigure 2. Mean Household Total Health Care Payments as a Share of Compensation Under Alternative Single-Payer Financing Assumptions eFigure 3. Percentage of Population Paying More or Less Relative to the Status Quo, by Income Group eReferences

Supplement 2. Data Sharing Statement
