# Supplementary material for: Household Health Care Payments Under Rate Setting, Spending Growth Target, and Single-Payer Policies
Source: JAMA Health Forum. 2024 Jun 30;5(6.9):e241932. doi: 10.1001/jamahealthforum.2024.1932 (PMC11215555; doi:10.1001/jamahealthforum.2024.1932)
Supplement: Supplement 1. — eMethods 1. RAND COMPARE Microsimulation Model eMethods 2. Adjustments to Health Care Expenditures and Payments eMethods 3. Calculating the Wage Pass Back eTable 1. Key Model Inputs and Assumptions eTable 2. Demographic Characteristics of the Synthetic Population, 2030 eTable 3. Estimated Wages in the Status Quo and Single-Payer Scenarios, 2030 eFigure 1. Estimated Total Health Care Expenditures Relative to the Status Quo under Alternative Assumptions eFigure 2. Mean Household Total Health Care Payments as a Share of Compensation Under Alternative Single-Payer Financing Assumptions eFigure 3. Percentage of Population Paying More or Less Relative to the Status Quo, by Income Group eReferences [file jamahealthforum-e241932-s001.pdf]

Supplemental Online Content

Liu JL, Girosi F, Lu R, Eibner C. Household health care payments under rate setting, spending growth target, and single-payer policies. *JAMA Health Forum*. 2024;5(6.9):e241932. doi:10.1001/jamahealthforum.2024.1932

eMethods 1. RAND COMPARE Microsimulation Model
eMethods 2. Adjustments to Health Care Expenditures and Payments
eTable 1. Key Model Inputs and Assumptions
eTable 2. Demographic Characteristics of the Synthetic Population, 2030
eTable 3. Estimated Wages in the Status Quo and Single-Payer Scenarios, 2030
eFigure 1. Estimated Total Health Care Expenditures Relative to the Status Quo under Alternative Assumptions
eFigure 2. Mean Household Total Health Care Payments as a Share of Compensation Under Alternative Single-Payer Financing Assumptions
eFigure 3. Percentage of Population Paying More or Less Relative to the Status Quo, by Income Group

This supplemental material has been provided by the authors to give readers additional information about their work.

© 2024 Liu JL et al. *JAMA Health Forum.*

eMethods 1. RAND COMPARE Microsimulation Model

The RAND COMPARE microsimulation model uses a utility maximization approach to estimate how individuals in the model weigh costs and benefits of each available insurance option in health insurance enrollment decisions, using the following equation:[1]

$$U_i^\alpha = u(e_i^\alpha) - PRCT_i^\alpha - OOP_i^\alpha - \frac{1}{2} rVar(OOP_i^\alpha)$$

Each individual *i*'s utility (*U*) associated with health insurance option *α* reflected the value of health care consumed (*u(e)*) minus the costs associated with the option including premium contributions (*PRCT*), out-of-pocket (*OOP*) costs, and financial risk, where *r* is the coefficient of risk aversion. The value of health care consumed was a function of total expenditures (*e*), which we estimated using MEPS data, elasticities from the RAND Health Insurance Experiment, and payment rates assigned to the option. Because *e* reflected both the number of services consumed and payments for those services, the approach implied that health care was more valuable when payment rates are higher. This assumption enabled us to capture possible access constraints that may affect the utility of health care in scenarios in which payment is reduced.

The model estimates premiums for each health insurance risk group (firm-specific risk pools for large firms, small group, and nongroup including the Marketplaces) based on the estimated expenditures of people enrolled. Premium contributions were conditional on firm size and other characteristics for workers, reflected eligibility for premium tax credits for marketplace enrollees, and were set to zero for Medicaid. The model runs by allowing individuals to make health insurance decisions, calculating the resulting premiums that re-enter the utility maximization equation, and iterating to reach a steady state.

The model is calibrated to ensure that model outputs match observed enrollment and premiums in existing data. The calibration process made adjustments to a constant term included in the utility function to increase or decrease the value of specific health insurance options to ensure that model outputs match targets. We derived targets from the merged CPS-MEPS-EHBS dataset or, in some cases, from external sources, such as effectuated marketplace enrollment data published by CMS.

© 2024 Liuet al. *JAMA Health Forum.*

eMethods 2. Adjustments to Health Care Expenditures and Payments

To ensure that health care expenditures align with the NHEA across the full population, we made several adjustments to the MEPS-based health care expenditures assigned to individuals in the synthetic population, following the approach first introduced by Sing et al., 2006.[2] First, we excluded OOP payments for non-durable medical products (e.g., over-the-counter medications). Second, we assigned expenditures to institutionalized and active-duty military individuals. To institutionalized individuals, we assigned NHE expenditures in nursing care facilities and continuing care retirement communities. Further, we assigned estimated acute care expenditures of the institutionalized to the institutionalized and estimated expenditures for active-duty military and foreign visitors[3] to the active-duty military population. Third, we allocated the remaining NHE expenditures that are not included in MEPS, such as non-patient care revenues, to individuals. We generally assumed that these expenditures had the same distribution across the population as the MEPS-based expenditures, with the exception of some categories such as workers' compensation that we allocated in proportion to wages and public health activities that we allocated uniformly to the population.

Although aggregate health care payments equal aggregate health care expenditures, health care spending for care received by an individual is not always paid for by that individual. We allocated expenditures to types of payment – OOP payments, premium contributions (individual and employer), tax payments supporting health care programs, and other payments – through several steps. Although we reported outcomes only for the civilian, non-institutionalized population in this study, we accounted for the institutionalized and active-duty military populations in the analysis to align with NHEA spending categories.

For the status quo, we assigned NHE OOP expenditures to the civilian, non-institutionalized population by allocating the NHE amount in proportion to the MEPS-based OOP costs in COMPARE. In the reform scenarios, we kept constant the portion of NHE OOP expenditures that are not in MEPS and estimated the change in the remaining OOP payments based on changes in coverage, insurance plans, and provider payment levels.

Similarly, we aligned private premiums estimated in COMPARE to NHE private health insurance spending (less private spending for nursing care facilities) by keeping fixed the portion of NHE private expenditures that are not in MEPS and estimating changes in the COMPARE private premiums for ESI and nongroup insurance. We assigned Medicare Part B and D premiums based on income to individuals aged 65 and older; we assumed that the distribution of Medicare Advantage premiums would be similar and confirmed that the total assigned Medicare premium amount was similar to reported premiums in the 2022 Trustees Report.[4] We assigned civilian TRICARE enrollees an average premium for TRICARE Prime and Select that was weighted by enrollment in the plan types.[5,6]

To estimate federal personal income, state personal income, and payroll taxes, we used NBER's TAXSIM. We used Taxsim35 to calculate taxes in 2022, and inflated the amounts to 2030 using CPI-U. To estimate the amount of taxes going to health care programs, we allocated NHE Medicare hospital expenditures in proportion to Medicare payroll taxes; all other NHE federal government expenditures (including other federal programs, public health activity, and research, structures, and equipment; and less premium contributions for Medicare and TRICARE) in proportion to federal income taxes; and NHE state and local government expenditures in proportion to state income taxes.

To estimate other payments, we allocated NHE private non-patient revenue, research, structures, and equipment in proportion to federal plus state income taxes, and workers' compensation and worksite health payments in proportion to wages.

© 2024 Liuet al. *JAMA Health Forum.*

eMethods 3. Calculating the Wage Pass Back

In our single-payer scenario, we assumed that firms would stop offering coverage and transmit the savings back to workers in the form of increased wage and salary income. While the possibility that firms would return health insurance savings to workers is well-established in economic theory and consistent with empirical evidence,[7–10] it is less certain how firms would allocate these savings across the workforce. We assumed that firms would pass back wages based on workers' income, with the size of the pass back increasing with the probability of insurance take up. Specifically, we used the following approach:

- Using the CPS, we estimated a logit model that predicted employer insurance take up as a function of wage and salary income for workers at offering firms.
- For each worker offered coverage, we used the logit model to estimate $\hat{p}_i$, the probability of enrolling in employer coverage given wage and salary income.
- For each offering firm f, we calculated the total health employer premium contributions T_f that would be passed back under the single-payer scenario.
- For each worker i at firm f, we calculated the wage pass back amount (t_{if}) as follows:

$$t_{if} = T_f * \frac{\hat{p}_i}{\sum_{j=1}^{N_f} \hat{p}_j}$$

where N_f is the total number of workers at firm f.

Because $\hat{p}_i$ increases with wage and salary income, this approach results in larger pass back amounts for higher-income workers. However, we acknowledge that alternative approaches to allocating employer premium pass backs, such as an equal allocation across all workers, could affect our incidence estimates.

© 2024 Liuet al. *JAMA Health Forum.*

eTable 1. Key Model Inputs and Assumptions

	Inputs and Assumptions	Sources
Population	U.S. population by age, sex, and race/ethnicity from the Census Bureau's 2023 National Population Projections.	U.S. Census Bureau, 2023[11]
Income and wages	Person income in the 2022 CPS ASEC was adjusted if values were top coded, to align with 2022 aggregate total personal income in the BEA; household income by quintile was then adjusted to align with the household income distribution by quintiles estimated by the CBO. The same approach was used to adjust wages, first to aggregate total wages in the BEA and then to the household distribution of wages by quintiles estimated by the CBO. Income and wages were inflated to 2030 using the Consumer Price Index for Urban Consumers (CPI-U) projected by the CBO.	U.S. Bureau of Economic Analysis, 2023;[12] CBO, 2022;[13] CBO, 2023[14]
Health care expenditures	Individuals in the CPS ASEC were matched to individuals in the 2018-2019 MEPS (by age, sex, race/ethnicity, health status, income, and health insurance category); MEPS expenditures were aligned to aggregate NHEA projected total health care expenditures for 2030.	CMS, 2023[15]
Health insurance choice	Individuals and health insurance eligibility units decided health insurance enrollment by weighing the utility of available options, which were ESI, nongroup, Medicaid, and uninsured for those under age 65 (those aged 65 and over are assumed to be on Medicare) in all scenarios except the single payer scenarios.<br><br>Single payer: All individuals residing the U.S., including undocumented immigrants, were eligible for the single-payer plan.	Cordova et al., 2013[1]
Demand for health care services	People used less care when cost sharing was higher, e.g., those with 25% cost sharing spending about 20% less than those with free care.	Newhouse and Insurance Experiment Group, 1993[16]
Supply of health care services	Single payer: Providers reduced the supply of health care services when payment levels decreased. We assumed that 50% of new demand would be unmet due to supply constraints, based on our prior modeling work.	Liu et al., 2018;[17] Liu and Eibner, 2019[18]

© 2024 Liuet al. *JAMA Health Forum.*

	Inputs and Assumptions	Sources
Provider payment and prescription drug and device prices	Rate setting: Our base assumption was all-payer rate setting equal to 110% of Medicare rates, which we estimated as the all-payer average in the status quo based on published relative rates paid by private health insurance, Medicaid, and Medicare weighted by health care expenditures for hospital, physician, other professional, dental, and home health services; prescription drugs; and durable medical equipment. In sensitivity analyses, we assumed rates equal to 120% of Medicare rates.<br><br>Single payer: Our base assumption was single-payer rates equal to 110% of Medicare rates. In sensitivity analyses, we assumed rates equal to 120% of Medicare rates. We assumed that prescription drug and device prices were 90% of Medicare rates.	American Hospital Association, 2020;[19] KFF, 2019;[20] Zuckerman et al., 2021;[21] Roehrig, 2018;[22] Gagnon and Wolfe, 2015;[23] Cook, 2013[24]
Actuarial value	Single payer: Our base assumption was a single-payer payer plan with an actuarial value of 98%. In sensitivity analyses, we assumed an actuarial value of 80%.	By assumption. A 98% actuarial value means that, on average, people would pay 2% of total costs out-of-pocket. An 80% actuarial value is similar to a gold plan on the Affordable Care Act marketplaces.
Health plan administration	Single payer: Our base assumption was a single-payer administrative load equal to 7%, which was approximately the overall administrative load of Medicare fee-for-service and Medicare Advantage in 2021. In sensitivity analyses, we assumed an administrative load of 10%, which we estimated to be the approximate administrative load for Medicare Advantage and Part D plans.	CMS, 2023;[15] Medicare Trustees, 2023[25]
Tax exclusion of employer-sponsored insurance (ESI)	We estimated the value of the tax exclusion for ESI as the ESI premium (both the employer and employee contributions) multiplied by the marginal tax rates for federal income, state income, and federal payroll taxes. The aggregate value of the tax exclusion was treated as federal health care spending that is financed by general revenue, which we attributed as part of personal income tax revenue.	Rae et al., 2014[26]
Wage pass back	Single payer: We assumed that employers adjust wages if they no longer contribute to premiums. We assumed that 100% of employer premium contributions in the status quo would be passed back to workers in the form of wages. The estimated wages passed back to each worker was proportional to an estimated probability that the worker takes the employer-sponsored insurance given wages, with the probability estimated using a logistic regression.	Anand, 2017;[27] Sommers, 2005[28]

© 2024 Liuet al. *JAMA Health Forum.*

	Inputs and Assumptions	Sources
Financing	Single payer: Our base assumption was that the single-payer plan would be financed by a payroll tax and income tax. The payroll tax was a flat rate applied to wages with the aggregate payroll tax revenue equaling aggregate employer premium contributions in the status quo; the income tax applied to personal income such that the aggregate income tax revenue equaled the remaining necessary revenue and rates were set in proportion to the federal income tax schedule in the status quo. In sensitivity analyses, we made following alternative assumptions:<br>• Income tax in proportion to the federal income tax schedule in the status quo (applied to personal income)<br>• Flat income tax rate (applied to personal income)<br>• Flat payroll tax rate (applied to wages)	Many single-payer proposals do not include detailed financing plans. Some past proposals have specified the types of taxes but not the tax levels. E.g., the New York Health Plan contained a payroll and non-payroll tax (on taxable income not subject to the payroll tax);[29] the American Health Security Act (S. 1782, 113th Congress) of 2013 contained a graduated income tax (2.2 to 5.2%), surcharge for high earners (5.4%), payroll tax paid by employers (6.7%), and a securities transaction tax (0.02%).[30]

eTable 2. Demographic Characteristics of the Synthetic Population, 2030

	Weighted Population, Millions (%)
**Age**	
0-17	69.6 (20%)
18-34	74.9 (22%)
35-49	67.3 (20%)
50-64	58.5 (17%)
65+	69.3 (20%)
**Sex**	
Female	172.5 (51%)
Male	167.0 (49%)
**Race and ethnicity**	
White, Non-Hispanic	190.5 (56%)
White, Hispanic	62.0 (18%)
Black	46.3 (14%)
Asian	23.9 (7%)
Other	16.8 (5%)
**Household income as percent of FPL**	
<139% FPL	70.7 (21%)
139-400% FPL	122.5 (36%)
401-1000% FPL	110.6 (33%)
>1000% FPL	35.8 (11%)

FPL= federal poverty level
NOTE: This table reports data for the US civilian, non-institutionalized population.

eTable 3. Estimated Wages in the Status Quo and Single-Payer Scenarios, 2030

	Status Quo	Single Payer	Percent Change
Total wages, billions	$13,640	$15,082	11%
Average wage by poverty category			
<139% FPL	$27,178	$29,816	10%
139-400% FPL	$45,090	$51,486	14%
401-1000% FPL	$77,443	$86,419	12%
>1000% FPL	$231,430	$240,662	4%

FPL= federal poverty level

In the single-payer scenario, we assumed that there would no longer be employer premium contributions. As employer premium contributions as part of workers' compensation package, we assumed that employers passed back wages to their workers who were no longer forgoing wages for health care benefits. We assumed that aggregate amount of employer premium contributions per employer would be passed back to their workers in proportion to an estimated probability of taking employer-sponsored insurance. eTable 3 shows estimated wages in the status quo and single-payer scenarios.

© 2024 Liuet al. *JAMA Health Forum.*

eFigure 1. Estimated Total Health Care Expenditures Relative to the Status Quo under Alternative Assumptions

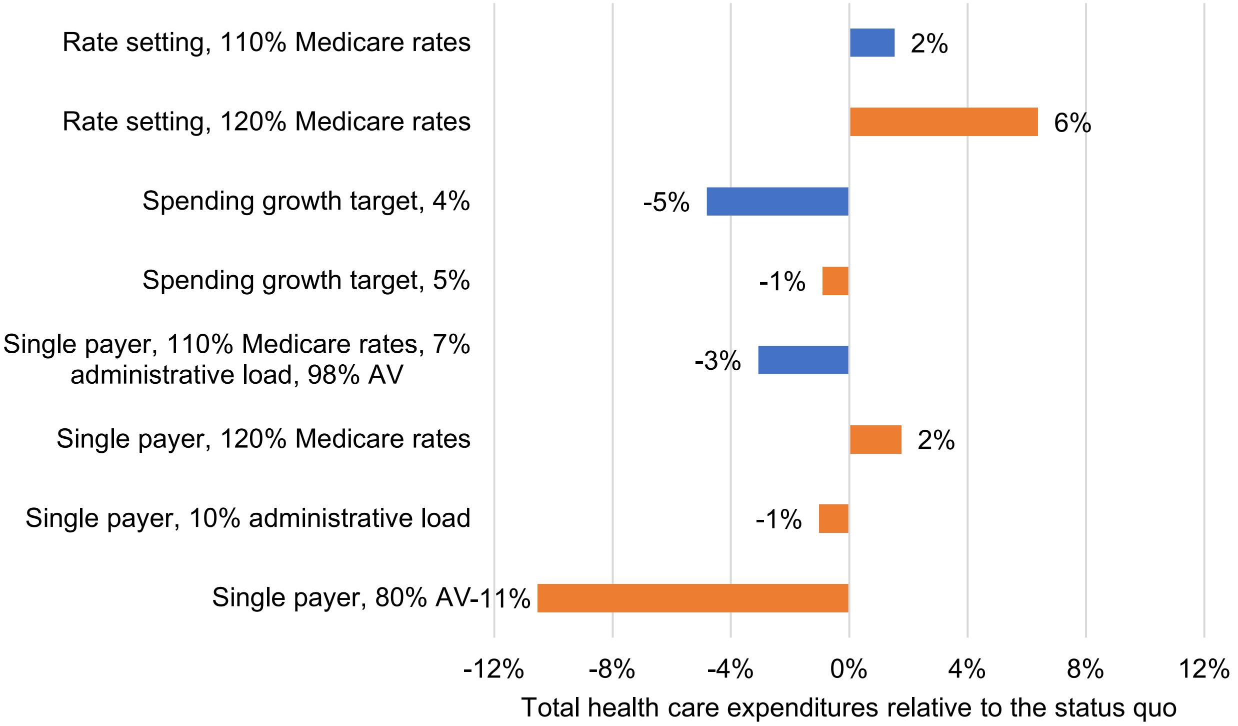

eFigure 1 shows the estimated total health care spending relative to the status quo for the scenarios presented in the main study (in blue) and for scenarios with alternative assumptions (in orange). For rate setting, increasing the all-payer rate setting level to 120% of Medicare rates increased total spending to 6% higher than total spending in the status quo. For spending growth targets, increasing the annual per capita growth rate to 5% increased total spending to 1% lower than total spending in the status quo. For the single payer scenarios, we varied one key assumption at a time compared to the single payer base scenario (110% Medicare rates, 7% administrative load, and 98% actuarial value). Increasing the single payer provider payment to 120% Medicare rates increased total spending to 2% higher than total spending in the status quo. Increasing the single payer administrative load to 10% increased total spending to 1% lower than total spending in the status quo. Decreasing the single payer actuarial value to 80% decreased to total spending to 11% lower than total spending in the status quo (note that this scenario substantially increases household spending for households with income below 139% FPL, as cost sharing is notably higher with the lower actuarial value; data not shown).

© 2024 Liuet al. *JAMA Health Forum.*

eFigure 2. Mean Household Total Health Care Payments as a Share of Compensation Under Alternative Single-Payer Financing Assumptions

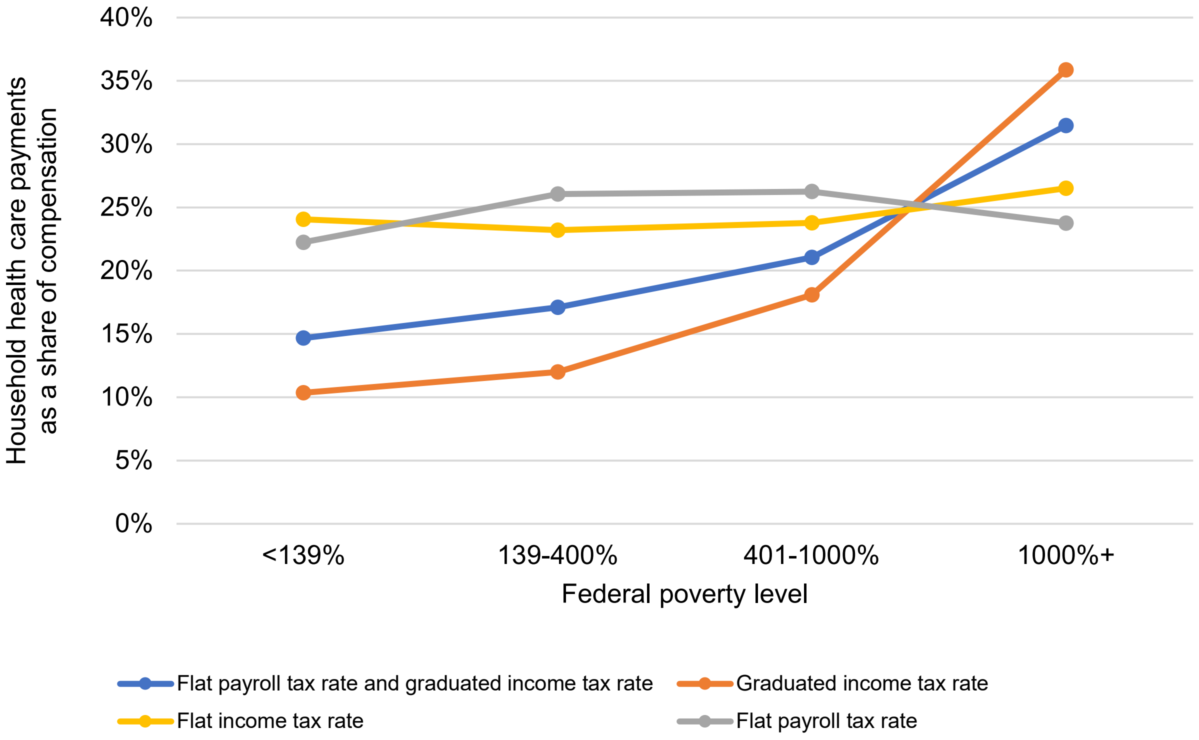

eFigure 2 shows the progressivity (or regressivity) of mean household total health care payments as a share of compensation across household income under alternative financing assumptions for the single payer base scenario (110% Medicare rates, 7% administrative load, and 98% actuarial value). Shown in blue is the financing structure presented in the main study, with a flat payroll tax rate on wages (that generates revenue equal to aggregate employer premium contributions in the status quo, which we estimated to be 9.7%) and a graduated income tax rate on personal income based on the federal income tax schedule in the status quo. A more progressive financing structure would be a graduated income tax rate alone (shown in orange; still based on the federal income tax schedule in the status quo). More regressive financing structures would be to have a flat income tax rate (shown in yellow; we estimated the rate to be 15.9%) or a flat payroll tax rate (shown in gray; we estimated the rate to be 26.7%).

eFigure 3. Percentage of Population Paying More or Less Relative to the Status Quo, by Income Group

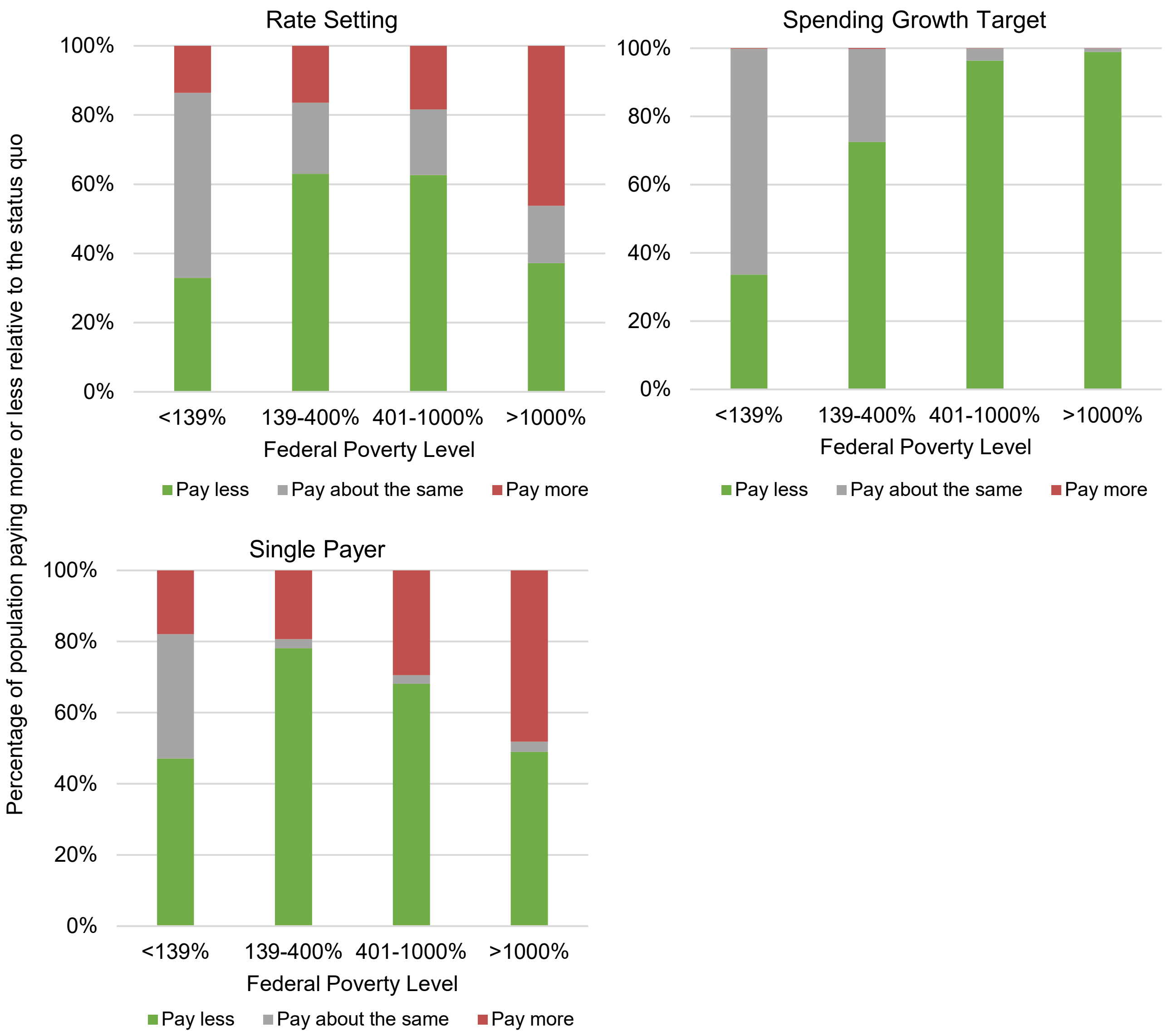

NOTE: This figure reflects payments made by the civilian, non-institutionalized population. The x-axis shows household income as a percent of the FPL in the status quo. Those who pay about the same either pay less than $100 in total health care payments or less than a 2% difference compared to the status quo.

eFigure 3 shows the percentage of the population paying more, less, or about the same under each reform scenario relative to the status quo. There was variability in how payments change for people within an income group. For example, while payments declined on average for the income groups except the highest income group reported in the single-payer scenario, there were people for whom payments increased in those lower-income groups, due to increased tax payments. In the rate setting scenario, some people paid more partly due to increased taxes with the higher Medicaid payment rates and enrollment, with 14% of people in households with income below 139% FPL paying more and 46% of people in households with income above 1000% FPL paying more. However, the majority pay less or about the same in the rate setting scenario. In the spending growth target scenario, nearly all people paid less or about the same compared to the status quo. People in higher-income households were more likely to pay less,

© 2024 Liuet al. *JAMA Health Forum.*

as they tended to have larger payments to start under current law and experienced larger effects with constrained spending growth. In the single-payer scenario, most people in households with income below 1000% FPL paid less or about the same; however, some people paid more due to more people being insured and increased taxes, with 18% of those below 139% FPL, 19% of those 139-400% FPL, and 29% of those 401-1000% FPL paying more. For those above 1000% FPL, 48% paid more under the single-payer scenario compared to the status quo.

© 2024 Liuet al. *JAMA Health Forum.*

eReferences

1. Cordova A, Girosi F, Nowak S, Eibner C, Finegold K. The COMPARE Microsimulation Model and the U.S. Affordable Care Act. *IJM*. 2013;6(3):78-117. doi:10.34196/ijm.00089
2. Sing M, Banthin JS, Selden TM, Cowan CA, Keehan SP. Reconciling medical expenditure estimates from the MEPS and NHEA, 2002. *Health Care Financ Rev*. 2006;28(1):25-40.
3. Bernard D, Cowan C, Selden T, Lassman D, Catlin A. *Reconciling Medical Expenditure Estimates from the MEPS and NHEA, 2012*. Agency for Healthcare Research and Quality; 2018. Accessed February 22, 2024. https://meps.ahrq.gov/data_files/publications/workingpapers/wp_17003.pdf
4. Boards of Trustees, Federal Hospital Insurance and Federal Supplementary Medical Insurance Trust Funds. *2022 Annual Report of the Boards of Trustees of the Federal Hospital Insurance Trust Fund and the Federal Supplementary Medical Insurance Trust Fund*.; 2022. Accessed February 29, 2024. https://www.cms.gov/files/document/2022-medicare-trustees-report.pdf
5. TRICARE Communications. Review Your TRICARE Health Plan Costs for 2022. TRICARE Newsroom. Published November 5, 2021. Accessed February 29, 2024. https://newsroom.tricare.mil/News/TRICARE-News/Article/2835574/https%3A%2F%2Fnewsroom.tricare.mil%2FNews%2FTRICARE-News%2FArticle%2F2835574%2Freview-your-tricare-health-plan-costs-for-2022
6. Health.mil. Patients by TRICARE Plan. Military Health System. Accessed February 29, 2024. https://www.health.mil/Military-Health-Topics/MHS-Toolkits/Media-Resources/Media-Center/Patient-Population-Statistics/Patients-by-TRICARE-Plan
7. Gruber J, Krueger AB. The Incidence of Mandated Employer-Provided Insurance: Lessons from Workers' Compensation Insurance. *Tax Policy and the Economy*. 1991;5:111-143. doi:10.1086/tpe.5.20061802
8. Blumberg LJ. Perspective: Who Pays For Employer-Sponsored Health Insurance? *Health Affairs*. 1999;18(6):58-61. doi:10.1377/hlthaff.18.6.58
9. Qin P, Chernew M. Compensating wage differentials and the impact of health insurance in the public sector on wages and hours. *Journal of Health Economics*. 2014;38:77-87. doi:10.1016/j.jhealeco.2014.08.001
10. Arnold D, Whaley C. Who Pays for Health Care Costs? The Effects of Health Care Prices on Wages. Published online July 21, 2020. doi:10.2139/ssrn.3657598
11. U.S. Census Bureau. 2023 National Population Projections Datasets. Census.gov. Published November 9, 2023. Accessed February 29, 2024. https://www.census.gov/data/datasets/2023/demo/popproj/2023-popproj.html
12. U.S. Bureau of Economic Analysis. SAINC4 Personal income and employment by major component. Published September 29, 2023. Accessed November 17, 2023. https://apps.bea.gov/itable/?ReqID=70&step=1&_gl=1*y7mu3z*_ga*OTAwNDQ0MjEzLjE3MDg2NDU1ODM.*_ga_J4698JNNFT*MTcwODY0NTU4NS4xLjEuMTcwODY0NTk0Mi42MC4wLjA.#eyJhcHBpZCI6NzAsInN0ZXBzIjpbMSwyOV0sImRhdGEiOltbIlRhYmxlSWQiLCI0OCJdXX0=
13. Congressional Budget Office. *The Distribution of Household Income, 2019*.; 2022. Accessed February 22, 2024. https://www.cbo.gov/publication/58353
14. Congressional Budget Office. Budget and Economic Data: Historical Data and Economic Projections. Published July 2023. Accessed February 22, 2024. https://www.cbo.gov/data/budget-economic-data#11
15. Centers for Medicare & Medicaid Services. National Health Expenditure Data. Published 2023. Accessed February 22, 2024. https://www.cms.gov/data-research/statistics-trends-and-reports/national-health-expenditure-data
16. Newhouse JP, Insurance Experiment Group. *Free for All? Lessons from the Rand Health Insurance Experiment*. Harvard University Press; 1993.
17. Liu JL, White C, Nowak SA, Wilks A, Ryan J, Eibner C. *An Assessment of the New York Health Act: A Single-Payer Option for New York State*. RAND Corporation; 2018. Accessed June 14, 2023. https://www.rand.org/pubs/research_reports/RR2424.html
18. Liu JL, Eibner C. *National Health Spending Estimates Under Medicare for All*. RAND Corporation; 2019. Accessed February 22, 2024. https://www.rand.org/pubs/research_reports/RR3106.html
19. American Hospital Association. *Trendwatch Chartbook 2020: Appendix 1, Supplementary Data Tables, Trends in the Overall Health Care Market*.; 2020. Accessed February 26, 2024. https://www.aha.org/system/files/media/file/2020/10/TrendwatchChartbook-2020-Appendix.pdf
20. KFF. Medicaid-to-Medicare Fee Index. Published 2019. Accessed February 26, 2024. https://www.kff.org/medicaid/state-indicator/medicaid-to-medicare-fee-index/

© 2024 Liuet al. *JAMA Health Forum.*

21. Zuckerman S, Skopec L, Aarons J. Medicaid Physician Fees Remained Substantially Below Fees Paid By Medicare In 2019. *Health Aff (Millwood)*. 2021;40(2):343-348. doi:10.1377/hlthaff.2020.00611
22. Roehrig C. Rebates, Coupons, PBMs, And The Cost Of The Prescription Drug Benefit. *Health Affairs Forefront.* Published April 26, 2018. Accessed February 26, 2024. https://www.healthaffairs.org/do/10.1377/forefront.20180424.17957/full/
23. Gagnon MA, Wolfe S. *Medicare Part D Pays Needlessly High Brand-Name Drug Prices Compared with Other OECD Countries and with U.S. Government Programs*. Carelton University and Public Citizen; 2015. https://carleton.ca/sppa/wp-content/uploads/Mirror-Mirror-Medicare-Part-D-Released.pdf
24. Cook A. Costs under Medicare's Prescription Drug Benefit and a Comparison with the Cost of Drugs under Medicaid Fee-for-Service. Presented at: AcademyHealth; June 23, 2013. https://www.cbo.gov/sites/default/files/cbofiles/attachments/44366_AcademyHealthPresentation_Cook.pdf
25. Boards of Trustees, Federal Hospital Insurance and Federal Supplementary Medical Insurance Trust Funds. *2023 Annual Report of the Boards of Trustees of the Federal Hospital Insurance Trust Fund and the Federal Supplementary Medical Insurance Trust Fund*.; 2023. Accessed April 17, 2024. https://www.cms.gov/oact/tr/2023
26. Rae M, Claxton G, Panchal N, Published LL. *Tax Subsidies for Private Health Insurance*. KFF; 2014. Accessed April 22, 2024. https://www.kff.org/report-section/tax-subsidies-for-private-health-insurance-i-federal-and-state-tax-exclusions-for-esi/
27. Anand P. Health Insurance Costs and Employee Compensation: Evidence from the National Compensation Survey. *Health Economics*. 2017;26(12):1601-1616. doi:10.1002/hec.3452
28. Sommers BD. Who Really Pays for Health Insurance? The Incidence of Employer-Provided Health Insurance with Sticky Nominal Wages. *Int J Health Care Finance Econ*. 2005;5(1):89-118. doi:10.1007/s10754-005-6603-5
29. Gottfried R. *New York Health Act*; A6058; New York Assembly; 2021-2022 Regular Session. Accessed April 17, 2024. https://www.nysenate.gov/legislation/bills/2021/A6058
30. Sanders B. *American Health Security Act of 2013*; S.1782; 113th Congress; 2013-2014. Accessed April 17, 2024. https://www.congress.gov/bill/113th-congress/senate-bill/1782

© 2024 Liuet al. *JAMA Health Forum.*
