# Supplementary material for: Household Health Care Payments Under Rate Setting, Spending Growth Target, and Single-Payer Policies
Source: JAMA Health Forum. 2024 Jun 30;5(6.9):e241932. doi: 10.1001/jamahealthforum.2024.1932 (PMC11215555; doi:10.1001/jamahealthforum.2024.1932)
Supplement: Supplement 2. — Data Sharing Statement [file jamahealthforum-e241932-s002.pdf]

Data Sharing Statement

Liu. Household Health Care Payments Under Rate Setting, Spending Growth Target, and Single-Payer Policies. *JAMA Health Forum*. Published June 30, 2024.
doi:10.1001/jamahealthforum.2024.1932

Data
Data available: Yes
Data types: Deidentified participant data
How to access data: All data are publicly available. CPS ASEC: https://www.census.gov/data/datasets/time-series/demo/cps/cps-asec.2022.html MEPS: https://meps.ahrq.gov/mepsweb/data_stats/download_data_files.jsp ACS PUMS: https://www.census.gov/programs-surveys/acs/microdata/access/2022.html KFF EHBS: https://www.kff.org/ehbs-data-use-form/
When available: With publication

Supporting Documents
Document types: None

Additional Information
Who can access the data: Anyone requesting the data for CPS ASEC, MEPS, and ACS PUMS. Researchers whose proposed use of the data has been approved for the KFF EHBS.
Types of analyses: For any purpose.
Mechanisms of data availability: Without investigator support.
